# Supplementary material for: Assessing the Functional Redundancy between P-gp and BCRP in Controlling the Brain Distribution and Biliary Excretion of Dual Substrates with PET Imaging in Mice
Source: Pharmaceutics. 2021 Aug 18;13(8):1286. doi: 10.3390/pharmaceutics13081286 (PMC8399697; doi:10.3390/pharmaceutics13081286)
Supplement: Supplementary file 1 [file pharmaceutics-13-01286-s001.zip › pharmaceutics-1322934-supplementary.pdf]

pharmaceutics

Supplementary Materials: Assessing the Functional Redundancy between P-gp and BCRP in Controlling the Brain Distribution and Biliary Excretion of Dual Substrates with PET Imaging in Mice

Irene Hernández-Lozano, Severin Mairinger, Alexander Traxl, Michael Sauberer, Thomas Filip, Johann Stanek, Claudia Kuntner, Thomas Wanek and Oliver Langer

Table S1. Area under the curve (AUC) values for blood, brain, liver and intestine time-activity curves for [11C]tariquidar, [11C]erlotinib and [11C]elacridar in wild-type, *Abcb1a/b*$^{(-/-)}$, *Abcg2*$^{(-/-)}$ and *Abcb1a/b*$^{(-/-)}$*Abcg2*$^{(-/-)}$ mice.

Radiotracer	AUC (%ID/mL x min)	Wild-type	*Abcb1a/b*$^{(-/-)}$	*Abcg2*$^{(-/-)}$	*Abcb1a/b*$^{(-/-)}$*Abcg2*$^{(-/-)}$
[11C]tariquidar	AUC_{blood}	403 ± 40	496 ± 51	641 ± 63	532 ± 36
	AUC_{brain}	45 ± 2	89 ± 3	55 ± 23	319 ± 58
	AUC_{liver}	1400 ± 67	1579 ± 67	1601 ± 77	1433 ± 92
	$AUC_{intestine}$	416 ± 48	507 ± 28	497 ± 33	393 ± 47
[11C]erlotinib	AUC_{blood}	326 ± 30	328 ± 26	359 ± 29	317 ± 29
	AUC_{brain}	62 ± 2	82 ± 7	81 ± 5	154 ± 7
	AUC_{liver}	1300 ± 152	1199 ± 106	1485 ± 101	1375 ± 93
	$AUC_{intestine}$	1173 ± 121	1023 ± 127	811 ± 67	622 ± 66
[11C]elacridar	AUC_{blood}	435 ± 72	455 ± 85	749 ± 203	469 ± 69
	AUC_{brain}	61 ± 13	99 ± 15	74 ± 7	243 ± 28
	AUC_{liver}	1388 ± 121	1351 ± 168	1672 ± 148	1362 ± 57
	$AUC_{intestine}$	390 ± 20	430 ± 35	584 ± 71	398 ± 28

AUC values are given as mean ± SD (n = 4–5 per group).

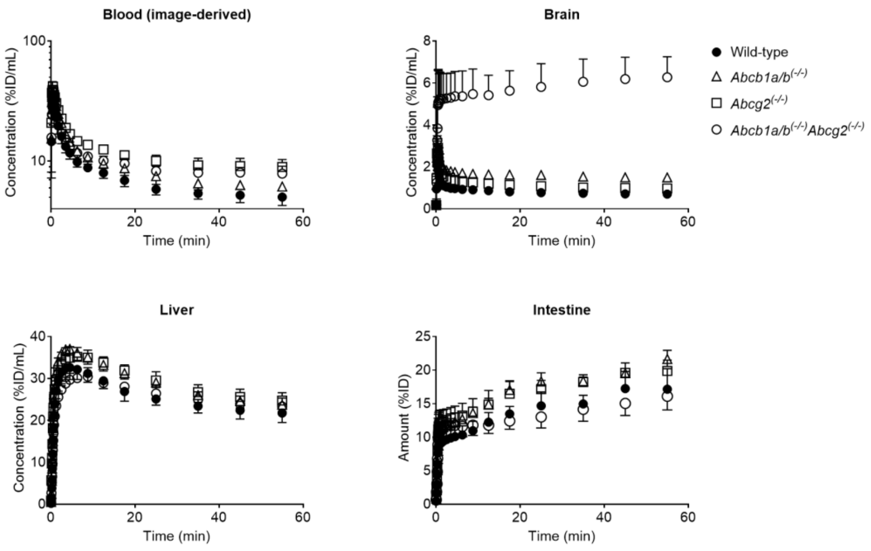

Figure S1. Mean time-activity curves (%ID/mL or %ID ± SD) of [11C]tariquidar in the blood (image-derived blood curve from the left ventricle of the heart), brain, liver and intestine in wild-type (•), *Abcb1a/b(−/−) (Δ)*, *Abcg2(−/−)* (□), and *Abcb1a/b(−/−)Abcg2(−/−)* (o) mice.

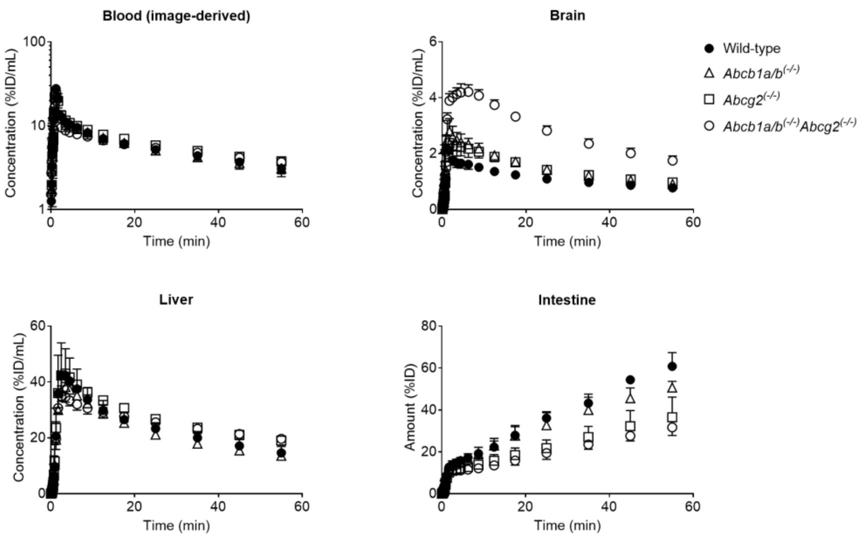

Figure S2. Mean time-activity curves (%ID/mL or %ID ± SD) of [11C]erlotinib in the blood (image-derived blood curve from the left ventricle of the heart), brain, liver and intestine in wild-type (•), *Abcb1a/b(−/−) (Δ)*, *Abcg2(−/−)* (□), and *Abcb1a/b(−/−)Abcg2(−/−)* (o) mice.

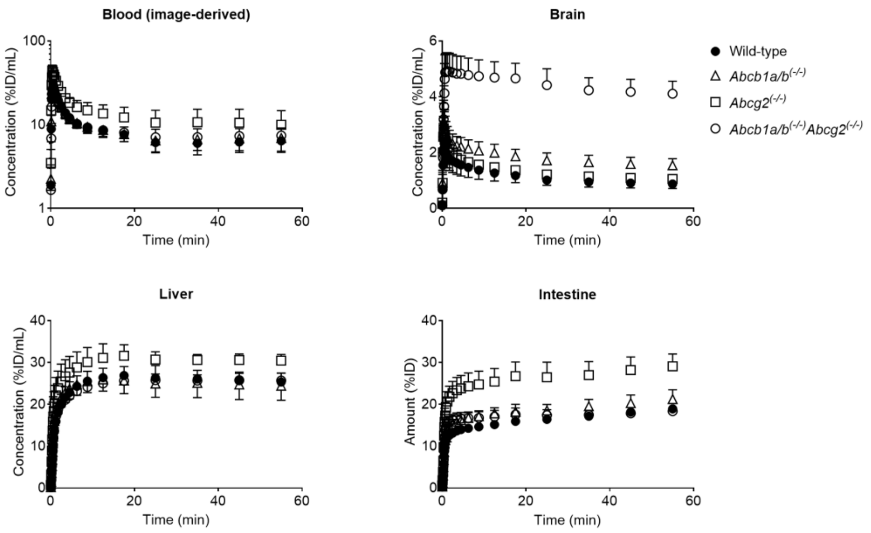

Figure S3. Mean time-activity curves (%ID/mL or %ID ± SD) of [11C]elacridar in the blood (image-derived blood curve from the left ventricle of the heart), brain, liver and intestine in wild-type (•), *Abcb1a/b(−/−)* (Δ), *Abcg2(−/−)* (□), and *Abcb1a/b(−/−)Abcg2(−/−)* (o) mice.

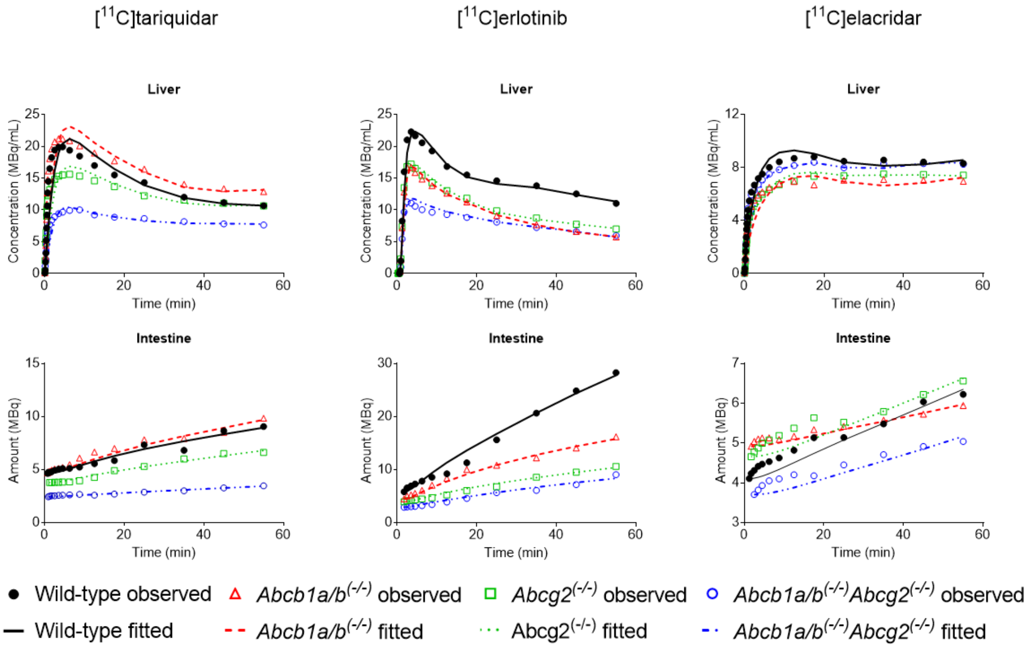

Figure S4. Observed and fitted time-activity curves (MBq/mL or MBq) of [11C]tariquidar, [11C]erlotinib and [11C]elacridar in the liver and intestine in one representative wild-type, *Abcb1a/b(−/−)*, *Abcg2(−/−)* and *Abcb1(−/−)Abcg2(−/−)* mouse.

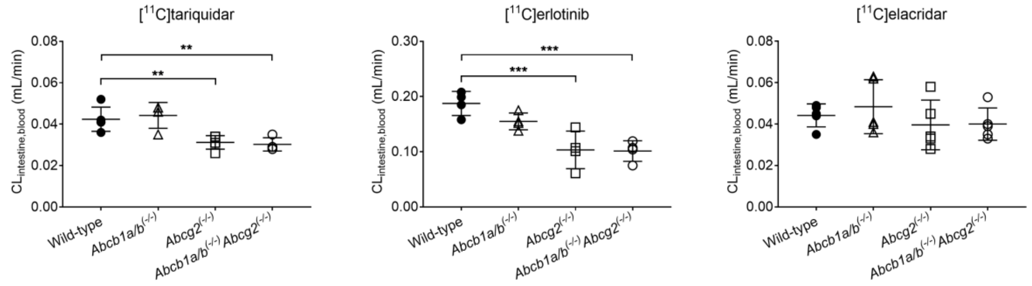

Figure S5. Intestinal clearance (CLintestine,blood) obtained by non-compartmental analysis for [11C]tariquidar, [11C]erlotinib and [11C]elacridar in wild-type, *Abcb1a/b(−/−)*, *Abcg2(−/−)* and *Abcb1a/b(−/−)Abcg2(−/−)* mice. ** $p \leq 0.01$, *** $p \leq 0.001$, one-way ANOVA followed by a Dunnett's multiple comparison test against the reference group (wild-type).
